# Bovine Mucosal Heparins Are Comparable to Porcine Mucosal Heparin at USP Potency Adjusted Levels

**DOI:** 10.3389/fmed.2018.00360

**Published:** 2019-01-09

**Authors:** Walter Jeske, Ahmed Kouta, Ambar Farooqui, Fakiha Siddiqui, Varun Rangnekar, Manoj Niverthi, Rajan Laddu, Debra Hoppensteadt, Omer Iqbal, Jeanine Walenga, Jawed Fareed

**Affiliations:** ^1^Cardiovascular Research Institute, Loyola University Chicago Health Sciences Division, Maywood, IL, United States; ^2^Georgia Thrombosis Forum, Suwanee, GA, United States

**Keywords:** heparin, potency, bovine, porcine, pharmacokinetics

## Abstract

**Introduction:** Bovine mucosal heparins (BMH) are currently being developed for re-introduction for both medical and surgical indications. BMH active pharmaceutical ingredient (API) exhibits a somewhat weaker USP potency when compared to PMHs. We hypothesized that when dosages are normalized based on the USP reference heparin, BMH will exhibit comparable *in vitro* and *in vivo* effects to those produced by PMH. Therefore, studies were developed to compare the APIs of bovine and porcine heparin.

**Materials and Methods:** API versions of PMH were obtained from Celsus Laboratories (Franklin, OH) and Medefil (Glen Ellen, IL). API versions of BMH were obtained from Kin Master (Passo Fundo, Brazil). Each of these heparins was assayed for their molecular weight profile, AT affinity, USP potency, and anticoagulant/antiprotease profiles using standard laboratory methods. *In vitro* protamine neutralization studies were carried out. Antithrombotic and hemorrhagic effects were measured in rats and pharmacodynamic profiles were assessed in primates.

**Results:** Size exclusion chromatography demonstrated that the mean molecular weight of BMH was ~15% higher than that of PMH (BMH: 20.1 ± 0.8 kDa and PMH: 17.5 ± 0.7 kDa). BMH exhibited an anti-Xa potency of 130 U/mg while PMH had an anti-Xa potency of 185 U/mg. In the anticoagulant and antiprotease assays, the BMH exhibited lower functionality which was proportional to USP potency. When the BMH was compared with PMH at potency adjusted concentrations, it showed identical concentration-response curves in the aPTT and anti-protease assays. However, in the protamine neutralization studies, BMH required slightly higher amounts of protamine in contrast to PMH. BMH and PMH administered to rats at equivalent anti-Xa unit dosages resulted in comparable antithrombotic activity and prolongation of bleeding time. Similar pharmacodynamic profiles were observed in primates when BMH and PMH were dosed on an anti-Xa U/kg basis.

**Conclusion:** BMH, when used at comparable anti-Xa unit levels, is comparable to PMH, however, it requires proportionally higher amount of protamine due to the increased mass for adjusting to higher potency. Additional studies on the structural characterization, interactions with PF4 and *in vivo* neutralization studies are ongoing.

## Introduction

Heparin products have long been used in the treatment of thrombotic conditions (1). The worldwide heparin market is approximately $7 billion per year, with more than half of this market consisting of low molecular weight (LMW) heparin (2). Even with the advent of new anticoagulants, it is anticipated that for the foreseeable future heparin and LMW heparin will remain standard clinical therapy and also a necessary component of successful surgeries and interventional procedures (1).

Heparin can be obtained in commercial quantities from intestinal mucosa (cow, pig, sheep) and lung (cow). Currently, porcine mucosal heparin is the primary source of raw material for heparin worldwide, and the only source for the most widely used LMW heparin enoxaparin. Following the heparin contamination crisis of a decade ago, and recognizing that the majority of world supply of porcine heparin originates from one country (3), there is interest from regulatory agencies in broadening the source of heparin (4). One means of accomplishing this would by the reintroduction of heparin derived from bovine tissue (5).

Heparins derived from different tissues and/or species have been shown to differ in their degrees of sulfation and acetylation, molecular weight, and anticoagulant activity (6–9). The chemical conditions under which heparins are isolated can also result in structural modifications and altered functional characteristics of the final product (10). Previous studies have demonstrated the similarity of unfractionated ovine and porcine heparins in terms of their structures and activities (11, 12). The correlation between structural differences and functional differences is not completely clear. It is known, however, that specific activities of bovine and porcine heparins in terms of their anti-Xa and anti-IIa activities are considerably different. The potency of bovine heparins is typically 30–50% less than that of porcine derived heparin (13). Such differences in activity may complicate the dosing of such drugs and may limit a clinician's ability to interchange different heparins.

Efforts have been made to develop bovine heparin preparations that are more similar to porcine heparin. One such attempt has been to treat bovine intestinal heparin with various sulfotransferases to increase the degree of 6-O and/or 3-O sulfation (13). Such modified heparins have been shown to have an increased number of antithrombin binding sites and anti-Xa and anti-IIa activities. Other studies have shown that via ion-exchange chromatography, it is possible to produce bovine heparin fractions enriched in 6-O sulfation that exhibit increased anticoagulant activity (14).

Earlier studies utilizing a rat vena caval thrombosis model (15) or clinical endpoint in patients undergoing hemodialysis (16) have demonstrated that dosing based on heparin international units results in equivalent endpoints. The current study compares the biologic activity of porcine and bovine heparins at equipotent concentrations or doses based on their USP anti-Xa potency.

## Materials and Methods

### Test Agents

This study utilized multiple lots of heparin derived from porcine intestinal mucosa (Celsus Laboratories, Franklin, OH and Medefil, Glen Ellen, IL) and bovine intestinal mucosa (Kin Master Indústrias Químicas, Passo Fundo, Brazil). The heparin samples were obtained as powders which were stored desiccated at room temperature. These samples were weighed on a Mettler balance and 0.9 % NaCl was used as a diluent to make stock solutions for the *in vivo* and *in vitro* testing.

### *In vitro* Studies

Molecular weight determinations were made using gel permeation chromatography (GPC) in a high-performance liquid chromatography (HPLC) system as previously reported (17). Briefly, the system was equilibrated using freshly degassed mobile phase (0.3 M sodium sulfate) until a stable baseline was obtained. Analysis was carried out by injecting 20 μl of sample (10 mg/ml in 0.3 M sodium sulfate; pH = 5.0) onto tandemly linked TSK G2000SW and TSK G3000SW columns (Tosoh Biosciences, Tokyo, Japan). The flow rate for the mobile phase was 0.5 ml/min and the run time for each sample was 65 min. The internal temperature for the RI detector was set at 35°C and UV determination was made at 205 nm. All analyses were made at room temperature. The elution profile of each sample was analyzed in relation to a calibration curve prepared using 13 heparin fractions ranging in molecular weight from 3.0 to 40 kDa. The molecular weight profile consisted of such parameters as weight average molecular weight, number average molecular weight, and polydispersity. The fraction of oligosaccharide chains with molecular weights < 8 kDa, 8–16 kDa, 16–24 kDa, and >24 kDa were determined from the slicing tables for each sample prepared using Millennium software (Waters, Milford, MA).

Porcine and bovine heparins were supplemented to normal human plasma over concentration ranges of 0.625 to 10 μg/ml or 0.0625 to 1 anti-Xa U/ml. Supplemented plasma samples were assayed for aPTT and anti-Xa and anti-IIa activities. aPTT measurements were made using TriniClot reagents (Tcoag, Wicklow, Ireland) (18) on an ACL ELITE instrument (Werfen, Bedford, MA). Anti-Xa and anti-IIa activities were determined using in-house amidolytic assays on the ACL ELITE (19). For the anti-Xa assay, bovine factor Xa (Enzyme Research Laboratories, South Bend, IN) was diluted in 50 mM Tris buffer (pH = 8.4) to a concentration of 1.25 IU/ml. Spectrozyme Xa (Biomedica Diagnostics, Windsor, Nova Scotia, Canada) was reconstituted in sterile water to make a 2.5 mM solution. The ACL ELITE was programmed to pipet 10 μl of plasma and 100 μl of factor Xa solution into a reaction rotor. Samples were incubated for 300 s before addition of 75 μl Spectrozyme Xa. Optical density at 405 nm was measured for 30 s. For the anti-IIa assay, human thrombin (Enzyme Research Laboratories, South Bend, IN) was diluted in 50 mM Tris buffer (pH = 8.4) to a concentration of 5 U/ml. Spectrozyme IIa (Biomedica Diagnostics, Windsor, Nova Scotia, Canada) was reconstituted in sterile water to make a 1 mM solution. The ACL ELITE was programmed to pipet 10 μl of plasma and 100 μl of thrombin solution into a reaction rotor. Samples were incubated for 120 s before addition of 40 μl Spectrozyme IIa. Optical density at 405 nm was measured for 30 s.

Anti-Xa and anti-IIa potencies were assessed using amidolytic assays from Aniara (West Chester, OH). Protamine sulfate was obtained from Sigma (St. Louis, MO).

### *In vivo* Studies

All animals were housed in accordance with the *Guide for the Care and Use of Laboratory Animals* (20). This study was carried out in accordance with the recommendations of Loyola University Chicago Health Sciences Division, Institutional Animal Care and Use Committee (IACUC). The protocol was approved by the IACUC.

### Rat Models

A rat jugular vein clamping model was used to assess antithrombotic activity (21). One batch each of bovine and porcine heparin was tested at doses ranging from 125 to 1,000 μg/kg and from 50 to 150 anti-Xa U/kg. Six rats per treatment group were evaluated. Briefly, following attainment of a sufficient plane of anesthesia with an intramuscular dose of ketamine (90 mg/kg) and xylazine (10 mg/kg), the skin on the neck was shaved. An incision was made centrally above the trachea and the right jugular vein was isolated. Baseline blood flow through the vessel was assessed using a bi-directional Doppler probe. Test heparins, or 0.9% NaCl vehicle, were administered via tail vein injection. After five minutes, the jugular vein is manually occluded using mosquito forceps. After one minute of occlusion, the forceps were released. Blood flow was again measured 5 min after release of the forceps. This procedure was repeated until the vessel had thrombosed as determined by no measurable blood flow 5 min after releasing the forceps. The effectiveness of the heparin treatment was quantified in terms of the number of clamping cycles required to cause vascular occlusion. Results are presented as mean ± SD.

Hemorrhagic activity was determined using a rat tail bleeding model (22). One batch each of bovine and porcine heparin was tested at doses ranging from 125 to 1,000 μg/kg and from 25 to 150 anti-Xa U/kg. Six rats per treatment group were evaluated. Briefly, following attainment of a sufficient plane of anesthesia with an intramuscular dose of ketamine (90 mg/kg) and xylazine (10 mg/kg), test heparins, or 0.9% NaCl vehicle, were administered via tail vein injection. Five minutes later, bleeding was induced by transection of the distal 2 mm of the rat tail using a scalpel blade. Free blood was gently blotted from the tail tip at 30 s intervals, taking care not to disrupt any clot, until bleeding stopped. Bleeding time was assessed as the time (in seconds) from tail transection to the cessation of bleeding. Results are presented as mean ± SD.

### Non-human Primate Model

Rhesus monkeys (*Macaca mulatta*) ranging in weight from 6.4 to 10.8 kg were used in this study (23). Primates were anesthetized by the intramuscular administration of ketamine (10 mg/kg) and xylazine (1–2 mg/kg) based on their most recent charted weight. Following attainment of the appropriate depth of anesthesia (assessed by a lack of response to foot pinch), primates were freshly weighed to accurately determine the dose of the test agent. The procedure room was maintained at an ambient temperature of 78°F to minimize the chance that primates would become hypothermic while under anesthesia. A baseline blood sample was collected by venipuncture of the saphenous vein. One batch each of bovine and porcine heparin was administered at a dose of either 0.5 mg/kg or 100 anti-Xa U/kg intravenously via the contralateral saphenous vein. Additional blood samples were collected at 15, 30, 60, and 120 min post-drug administration. Four primates were dosed with each heparin.

All blood samples were collected using a double syringe technique, employing a 21 gauge butterfly needle. After an initial ~1 ml volume (discard blood) was collected, the syringe was changed and a 2.7 ml sample was drawn and placed into a tube containing 0.3 ml 3.2% sodium citrate. Citrated blood samples were centrifuged at 1,100 x g for 15 min. The supernatant platelet poor plasma was harvested and aliquots of plasma were stored frozen at −70° until analysis of circulating drug levels.

*In vitro* concentration-response curves were made by supplementing the bovine and porcine heparins into pooled primate plasma. Plasma concentrations of the various heparins were plotted against corresponding optical densities in the factor Xa or factor IIa assays using graphing software, SigmaPlot for Windows version 12.3 (Systat Software, San Jose, CA) and best-fit curves were made. The drug concentration in each primate blood sample in terms of anti-Xa and anti-IIa activities was determined by extrapolation. The area under the plasma concentration time curve (AUC) was calculated from the extrapolated plasma concentrations using the PKSolver^®^ add-in software for Microsoft Excel (24). All results are presented as mean ± SD.

### Statistical Analysis

Statistical analysis was carried out using SigmaPlot for Windows, version 12.3 (Systat Software, San Jose, CA). Differences between bovine and porcine heparin were assessed by *t*-test if data were normally distributed and by the Mann-Whitney test if data were not normally distributed. Dose- and concentration-response curves for bovine and porcine heparins were analyzed by two-way ANOVA followed by the Holm-Sidak multiple comparison test. *p*-values ≤ 0.05 were considered statistically significant.

## Results

**Figure 1:**
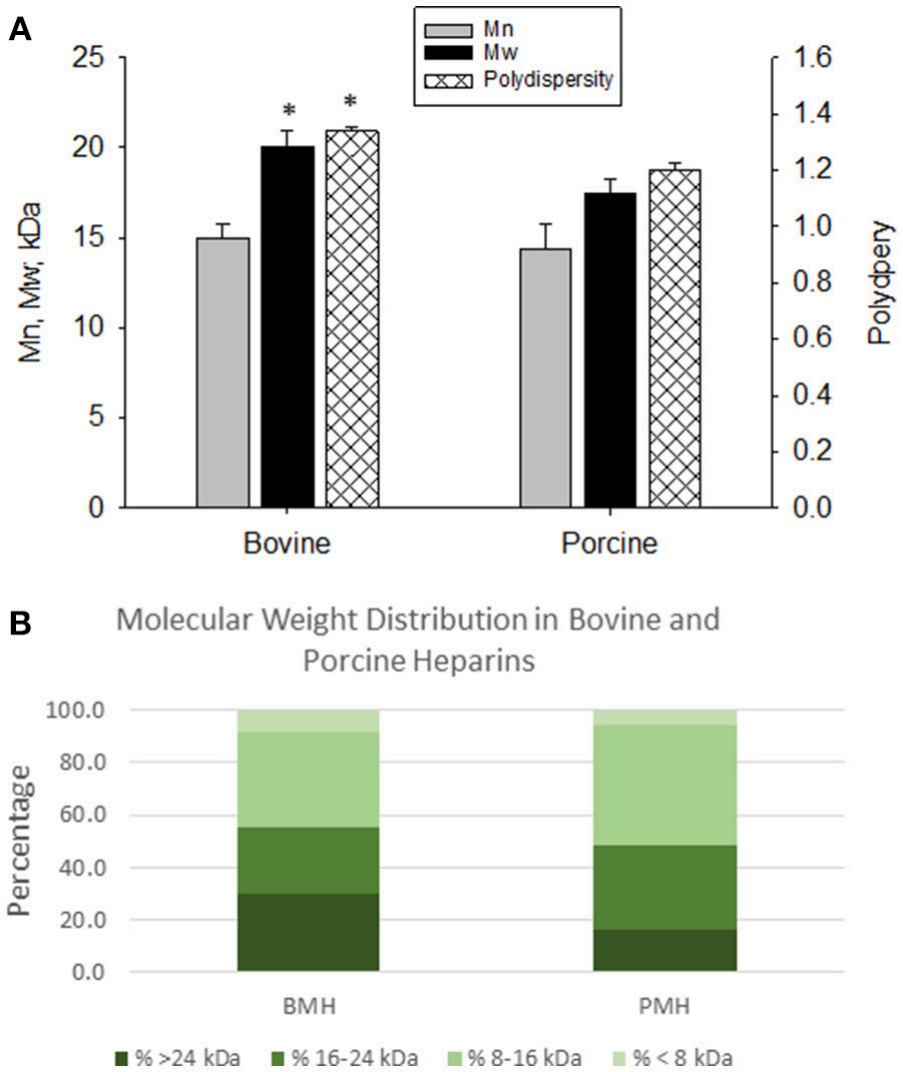
Molecular weight profiles of bovine and porcine heparin. The molecular weight profile of bovine (*n* = 16 batches) and porcine (*n* = 4 batches) heparins were assessed by size exclusion chromatography. **(A)** Bovine heparins exhibited a higher weight average molecular weight and polydispersity compared to porcine heparins. Results are presented as mean ± SD. **(B)** The fraction of oligosaccharide chains with molecular weight > 24 kDa was nearly two-fold higher in bovine heparin compared to porcine heparin samples (29.6 ± 3.1 % vs. 16.2 ± 3.1 %; *p < 0.001, *t*-test).

The elution profiles by size-exclusion, gel-permeation chromatography indicated good batch-to-batch reproducibility in terms of molecular weight profile. The bovine heparins exhibited a somewhat larger molecular weight compared to porcine heparins. Weight average molecular weight for bovine heparins averaged 20.1 ± 0.84 kDa vs. 17.5 ± 0.72 kDa for porcine heparins (*p* < 0.001). The number average molecular weight was numerically, but not significantly, lower for porcine heparins compared to bovine heparin. Polydispersity values were somewhat higher for the bovine heparins compared to porcine heparins (1.338 ± 0.018 vs. 1.198 ± 0.025; *p* < 0.001) (Figure 1A). Figure 1B shows the mean distribution of oligosaccharide components in bovine and porcine heparin samples. The percentage of oligosaccharide chains in the bovine heparin samples with molecular weight >24 kDa was observed to be nearly two-fold the percentage observed with the porcine samples (29.6 ± 3.1 % vs. 16.2 ± 3.1 %; *p* < 0.001, *t*-test).

**Figure 2:**
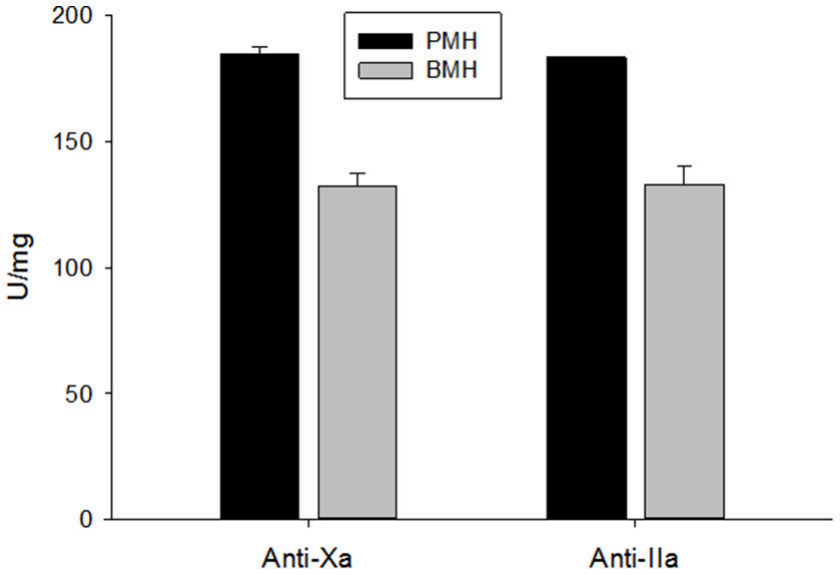
USP potency determined using amidolytic anti-Xa and anti-IIa assays. Bovine heparins exhibited potencies that were ~30% lower than those observed for porcine heparins. Results are presented as mean ± SD.

Potency of the bovine- and porcine-derived heparins was assessed relative to the USP heparin reference standard for assays using amidolytic assays (Figure 2). Porcine heparins exhibited mean anti-Xa and anti-IIa potencies of 184.6 ± 2.8 and 183.4 ± 1.7 U/mg, respectively. The potencies of bovine heparin were significantly lower at 132.4 ± 5.0 and 133.0 ± 7.5, respectively, based on anti-Xa and anti-IIa activity (*p* = 0.003 for anti-Xa and anti-IIa, Mann-Whitney test). It is this anti-Xa potency that was used to adjust dosing in subsequent studies.

**Figure 3:**
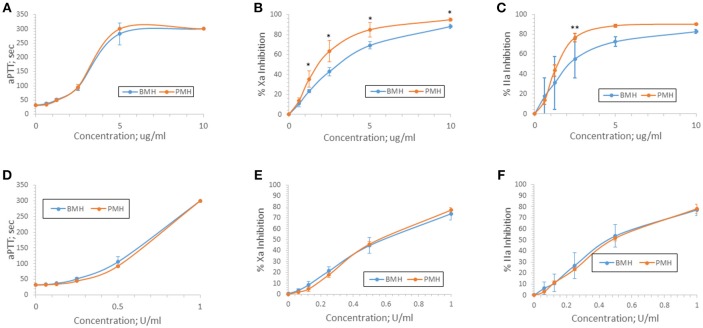
Anticoagulant and antiprotease activities of porcine and bovine heparin when supplemented to human plasma. Bovine (*n* = 16 batches) and porcine (*n* = 4 batches) were supplemented to human plasma at concentrations ranging from 0.625 to 10 μg/ml **(A–C)** or 0.0625 to 1.0 anti-Xa U/ml **(D–F)**. Supplementing heparins to plasma minimized the activity differences between bovine and porcine heparins. Results are presented as mean ± SD. *p < 0.001 PMH vs. BMH; **p = 0.010 PMH vs. BMH, two-way ANOVA.

Anticoagulant activity of bovine and porcine heparins was assessed using aPTT, anti-Xa, and anti-IIa assays following supplementation to normal human plasma at concentrations up to 10 μg/ml or 1 anti-Xa U/ml. When bovine and porcine heparins were supplemented at equigravimetric concentrations, bovine heparin produced weaker anti-Xa and anti-IIa effects compared to porcine heparin. Surprisingly, this difference was not reflected in the prolongation of aPTT by bovine and porcine heparins (Figures 3A–C). When heparins were instead supplemented on the basis of anti-Xa unit activity, the concentration-response curves for the amidolytic anti-Xa and anti-IIa assays for bovine and porcine heparin were nearly superimposable (Figures 3D–F).

**Figure 4:**
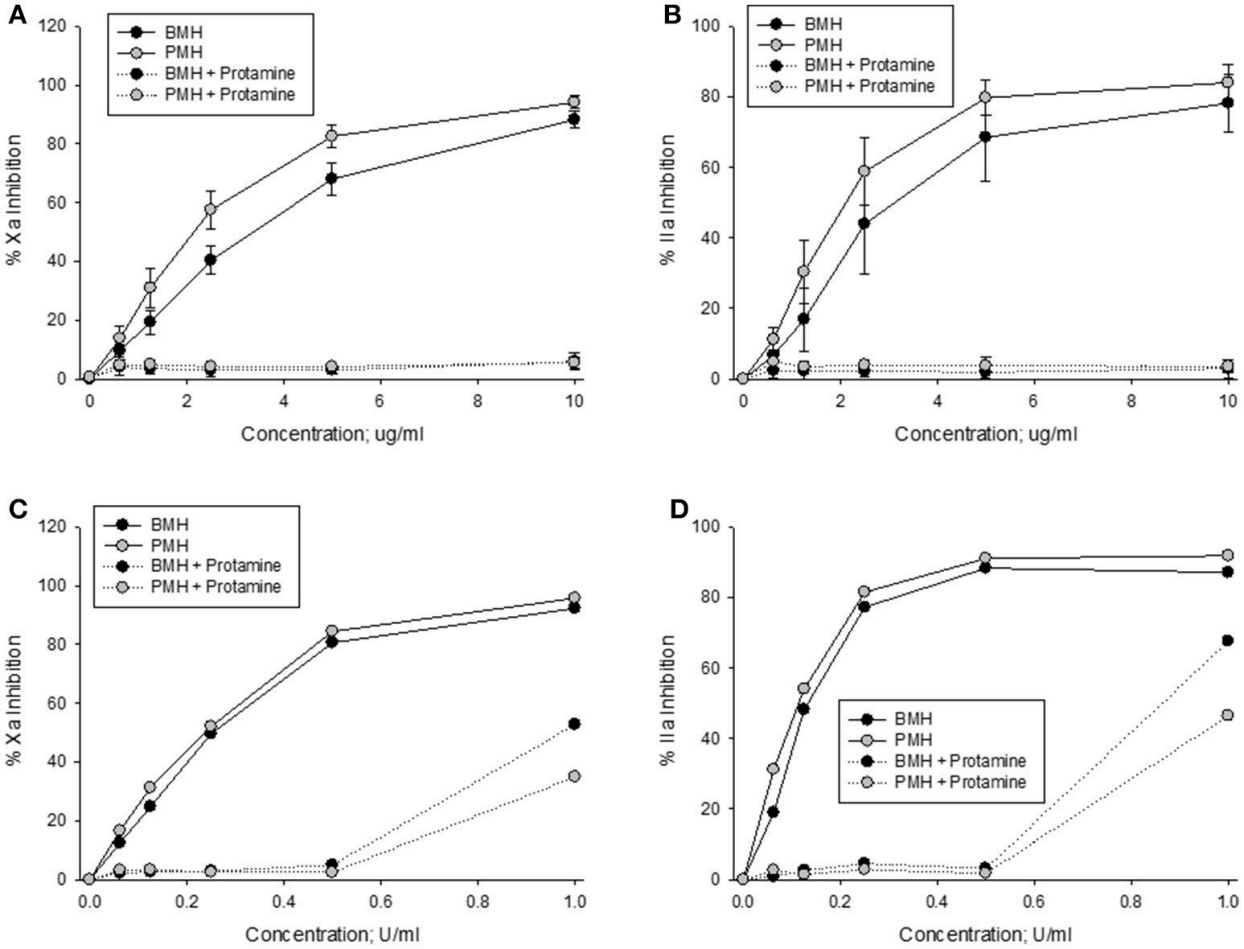
*In vitro* protamine neutralization of porcine and bovine heparin as measured by the anti-Xa **(A,C)** and anti-IIa **(B,D)** assays. When heparins were supplemented to plasma on an equal unit basis **(C,D)**, protamine was less able to neutralize bovine heparin.

Anti-Xa and anti-IIa activities of bovine and porcine heparins at concentrations up to 10 μg/ml were completely neutralized by a fixed protamine concentration of 10 μg/ml (Figures 4A,B). In contrast, when heparins were supplemented to plasma in anti-Xa U/ml concentrations, there was a higher level of residual (not neutralized) activity in the bovine heparin supplemented plasma samples (Figures 4C,D).

**Figure 5:**
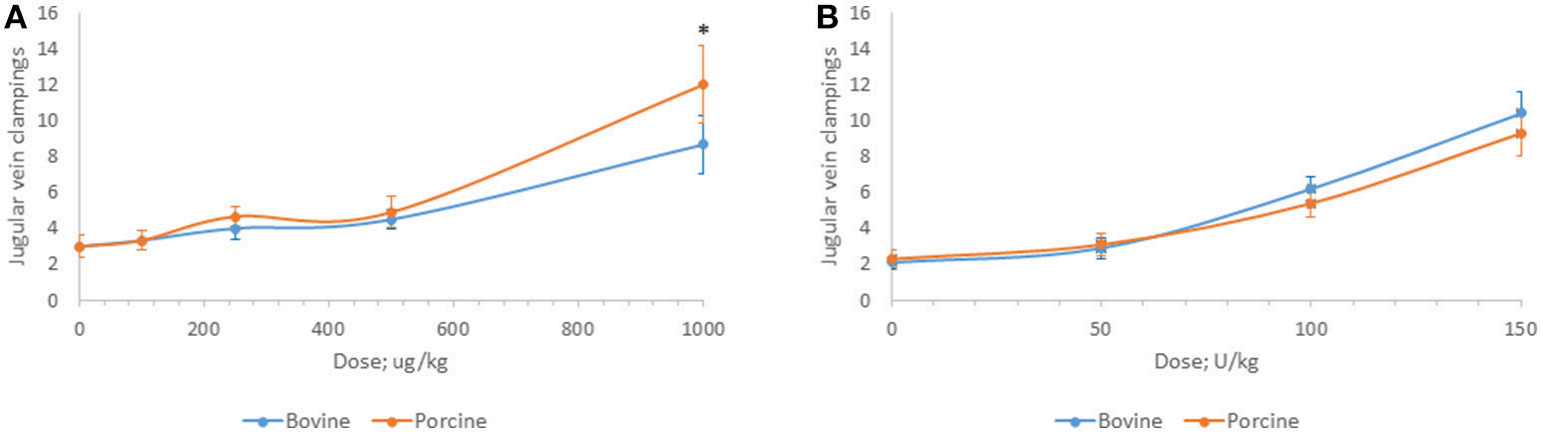
Antithrombotic activity of bovine and porcine heparin in a rat model. Bovine and porcine was administered intravenously to groups of rats (*n* = 6/dose) at doses ranging from 100 to 1,000 μg/kg **(A)** or 50 to 150 anti-Xa U/kg **(B)**. *p < 0.001 PMH vs. BMH, two-way ANOVA.

**Figure 6:**
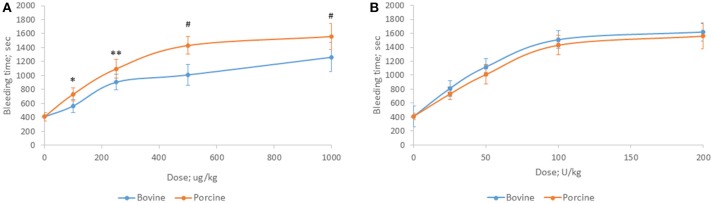
Hemorrhagic activity of bovine and porcine heparin in a rat model. Bovine and porcine was administered intravenously to groups of rats (*n* = 6/dose) at doses ranging from 100 to 1,000 μg/kg **(A)** or 25 to 200 anti-Xa U/kg **(B)**. *p = 0.033, **p = 0.016, ^#^*p* < 0.001 PMH vs. BMH, two-way ANOVA.

Bovine and porcine heparins were administered to animals to assess the impact of potency-based dosing on their biologic activity. A rat model of jugular vein damage was chosen to assess antithrombotic activity. Consistent with its weaker anticoagulant activity measured *in vitro*, fewer jugular vein clamping cycles were required to reach jugular occlusion in rats treated with higher dosages of bovine heparin (Figure 5A). At a dose of 1,000 μg/kg, rats treated with porcine heparin required 12.1 ± 1.3 clampings compared to 8.8 ± 0.9 clampings in bovine heparin-treated rats (*p* < 0.001). When heparins were administered over a range of 50 to 150 anti-Xa U/kg, the differences between heparins were minimized and bovine heparin-treated animals exhibited slightly higher, but not statistically different, antithrombotic activity compared to porcine heparin-treated animals (Figure 5B). A similar pattern of activities was observed when prolongation of bleeding time was assessed. When dosed on a gravimetric basis, porcine heparin produced statistically significantly longer bleeding times at all doses tested compared to bovine heparin treatment (Figure 6A). When bovine and porcine heparins were dosed at equivalent anti-Xa U/kg levels, similar prolongations in bleeding time were observed with both heparin treatments (Figure 6B).

**Figure 7:**
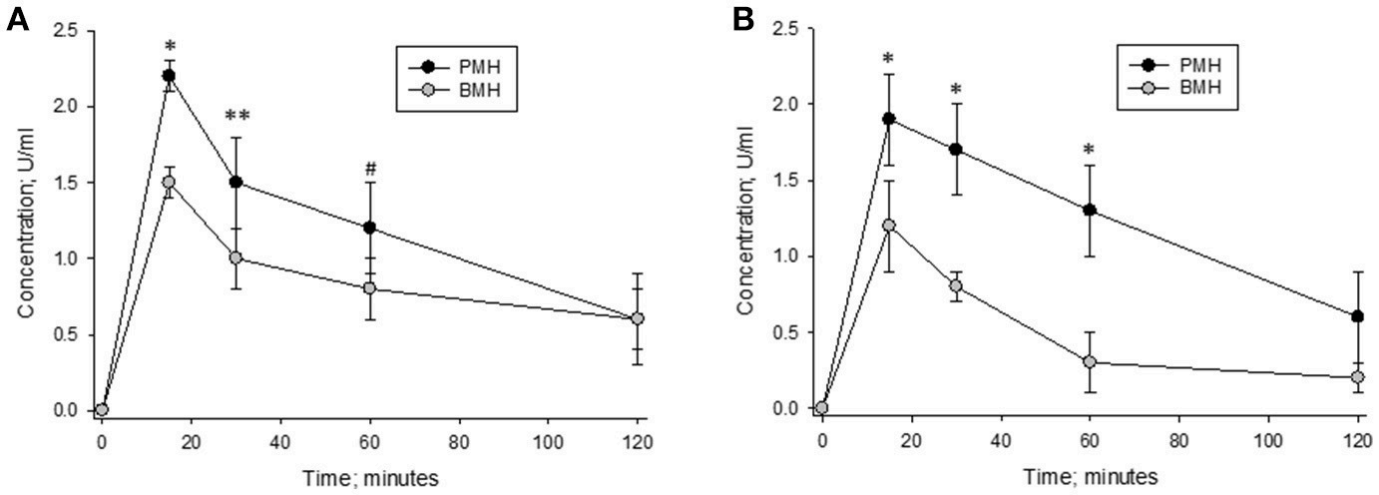
Pharmacodynamic effects of a 500 μg/kg intravenous dose of bovine and porcine heparin. Bovine and porcine was administered intravenously to groups of primates (*n* = 4/heparin). Plasma heparin concentrations determined using anti-Xa **(A)** and anti-IIa **(B)** assays are reported as mean ± SD. *p < 0.001, **p = 0.003, ^#^*p* = 0.006 PMH vs. BMH, two-way ANOVA.

Heparins were administered intravenously to non-human primates in order to measure their pharmacodynamic effects (Figure 7). Multiple blood samples were collected over a period of 2 h post-administration and circulating heparin concentrations in U/ml were extrapolated from *in vitro* anti-Xa and anti-IIa calibration curves. When heparins were administered at a dose of 500 μg/kg, lower circulating levels were observed in bovine heparin-treated animals compared to those treated with porcine heparin. Peak levels measured at 15 min post-administration were approximately 30% lower in bovine heparin-treated animals than in those receiving porcine heparin. By anti-Xa assay, peak levels were 1.53 ± 0.12 and 2.2 ± 0.1 U/ml (*p* < 0.001), respectively, in bovine and porcine heparin-treated animals. A similar difference was observed when heparin levels were determined by anti-IIa activity (1.23 ± 0.12 and 1.87 ± 0.24 U/ml (*p* < 0.001), respectively, in bovine and porcine heparin-treated animals). When anti-Xa activity was used to determine plasma concentrations, the ratio of AUCs in bovine and porcine treated primates was 0.70 (113.3 ± 21.6 vs. 162.5 ± 25.8 U^*^min^*^ml^−1^; *p* = 0.026), comparable to the ratio of anti-Xa potencies determined *in vitro*. When anti-IIa activity was used to determine plasma concentrations, a larger difference in ratio of AUCs (ratio = 0.60) was observed (95.4 ± 6.9 vs. 157.9 ± 27.8 U^*^min^*^ml^−1^; *p* = 0.005).

**Figure 8:**
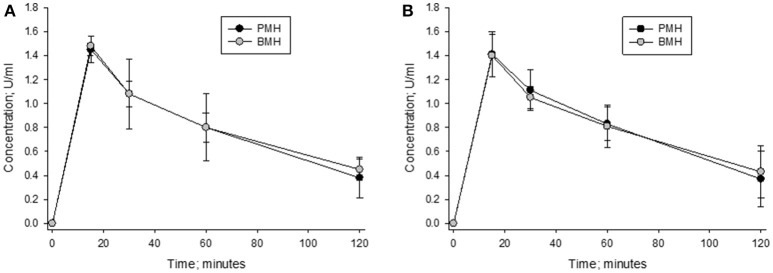
Pharmacodynamic effects of a 100 anti-Xa U/kg intravenous dose of bovine and porcine heparin. Bovine and porcine was administered intravenously to groups of primates (*n* = 4/heparin). Plasma heparin concentrations determined using anti-Xa **(A)** and anti-IIa **(B)** assays are reported as mean ± SD.

The concentration vs. time curves for porcine and bovine heparins were nearly superimposable when concentrations were measured using either anti-Xa or anti-IIa assays following administration of 100 anti-Xa U/kg doses (Figure 8). Peak levels of 1.48 ± 0.09 and 1.45 ± 0.13 anti-Xa U/ml were observed in bovine- and porcine-treated animals, respectively. By anti-IIa assay, peak levels of 1.40 ± 0.21 and 1.41 ± 0.22 U/ml were observed in bovine- and porcine-treated animals, respectively. The AUC values determined using circulating drug levels based on anti-Xa and anti-IIa activities were also comparable. Using drug levels determined by anti-Xa assay, AUCs for bovine and porcine heparin treated animals were calculated to be 111.5 ± 11.0 and 108.8 ± 26.7 U^*^min^*^ml^−1^, respectively. Using drug levels determined by anti-IIa assay, AUCs for bovine and porcine heparin treated animals were calculated to be 108.5 ± 24.2 and 108.1 ± 23.5 U^*^min^*^ml^−1^, respectively.

## Discussion

Despite the development of orally available, specific factor Xa and thrombin inhibitors, heparin and its low molecular weight derivatives remain critical drugs for the prevention and treatment of thrombotic conditions (1). Although bovine-derived heparins are still in use in some countries due to cultural restrictions on consumption of porcine-derived materials (25), the outbreak of bovine spongiform encephalopathy in the 1990s has led to the exclusive use of porcine heparin in Western countries. The heparin contamination crisis of 2007–2008 has focused attention on the need to diversify the heparin supply chain. The reintroduction of bovine heparin to the US market is viewed by the US FDA as a means to prevent supply interruptions (5).

It is established that bovine heparins exhibit lower anticoagulant activity than porcine heparin (9, 13, 14, 25, 26) Modern analytical techniques have shown this reduced activity of bovine heparin to be associated with differences in the sulfation pattern and presence of antithrombin-binding regions (6, 12, 13, 27, 28). Even with modern refinements to the heparin manufacturing process, the potency of bovine heparins will likely be limited to ~75% that of porcine heparin. Attempts to get around this limit have included sulfonation and fractionation of bovine heparins (13, 14). It remains to be seen whether such processes can be economically viable.

Administering a greater amount of bovine heparin by weight, and thereby administering similar amounts of anticoagulant activity, may be necessary to facilitate clinical acceptance and use of heparins derived from two different sources. It has recently been shown that use of the 6th International Standard for Unfractionated Heparin, which is a porcine heparin, is suitable for assessing potency of bovine heparins (29). In the current study, we utilized the USP heparin activity standard to normalize concentrations of bovine and porcine heparin for *in vitro* assessment of anticoagulant activity, and doses of these heparins for use in *in vivo* models.

Consistent with previous reports (9, 13, 14, 25, 26), it was shown that the bovine heparins studied here exhibited lower anti-Xa and anti-IIa potencies (approximately 130 U/mg) compared to porcine heparins (~185 U/mg). Our data show that this reduced potency of bovine heparins was associated with reductions in anticoagulant and antithrombotic and hemorrhagic effects in animal models. Supplementing or administering these heparins at equivalent anti-Xa concentrations or doses minimized most of these differences. This was clearly seen in the pharmacodynamic studies carried out in primates where the concentration vs. time curves were superimposable for bovine and porcine heparins administered at a dose of 100 anti-Xa U/kg. These findings support the results of a small clinical study in which patients undergoing cardiac bypass surgery were randomized to receive either bovine mucosal heparin or standard porcine heparin (30). In this study, total doses of bovine and porcine heparin in terms of units differed by ~2% and patients exhibited comparable prolongations of activated clotting time and amounts of post-surgical blood loss.

An exception to the above general trend relates to protamine neutralization. *In vitro*, it was shown that at a fixed dose of protamine, more residual anti-Xa and anti-IIa activity (less neutralization) was observed with bovine heparin. This is consistent with previous findings (29) where it was demonstrated that 1 mg of protamine could precipitate a greater amount of porcine heparin by activity compared to bovine heparin. The same study showed that there was a greater amount of residual (non-neutralized) activity in bovine heparin treated plasma compared to plasma porcine heparin treated plasma, when heparin activity was measured using aPTT, AT-dependent anti-Xa and anti-IIa and HC-II-dependent anti-IIa assays. Studies are ongoing to determine the extent to which such differences exist *in vivo* following administration of protamine to animals anticoagulated with bovine or porcine heparin. In the study by Gomes (30), the dose of protamine administered to patients receiving bovine heparin was numerically, but not statistically significantly, higher than the dose administered to porcine heparin-treated patients. While it appears that administering bovine and porcine heparins on an activity-basis may be a promising means to allow interchange these drugs, it is important to remember that potency assessment of heparins only characterizes their antithrombin-mediated activities. Heparin chains lacking the antithrombin-binding pentasaccharide are still able to inhibit thrombin via heparin cofactor II and can release TFPI. It has been shown that while potencies determined for porcine heparin using AT and HC-II-dependent assays are comparable, bovine heparins exhibit a higher HC-II-anti-IIa potency than AT-anti-IIa potency (165 IU/mg vs. 107 IU/mg) (29). Both effects may increase the overall amount of anticoagulant activity produced by bovine heparins. A safety concern with using heparins is the development of heparin-induced thrombocytopenia. It has been shown that particular ratios of heparin to platelet factor 4 are required to produce complexes that are antigenic (31). Administration of bovine and porcine heparins on an equi-unit basis will lead to different circulating molar concentrations of bovine and porcine heparins that may result in different antigenicity. Preliminary data using an *in vitro* platelet aggregation assay suggest that while bovine and porcine heparins can cause similar levels of platelet aggregation in the presence of HIT serum, they do so over different ranges of concentrations (data not shown). Such a finding may be related to the size of the complexes formed between platelet factor 4 and bovine or porcine heparin. A recent study utilizing photon correlation spectroscopy and zeta sizing techniques has shown that bovine mucosal heparin forms smaller complexes with platelet factor 4 than does porcine heparin at equivalent platelet factor 4:heparin molar ratios (32). The literature is unclear as to whether bovine heparins exhibit different antigenicity than porcine heparins when administered to human patients (33, 34).

These preliminary studies suggest that anti-Xa potency equated doses of bovine and porcine mucosal heparins may exhibit similar anticoagulant, antithrombotic and hemorrhagic effects. Further studies of the *in vivo* neutralization of anti-Xa potency equated doses of bovine and porcine mucosal heparins are warranted.

## Author Contributions

WJ, DH, and JF contributed to the conception and design of the study. AF, AK, FS, VR, MN, RL, and OI performed the laboratory analyses. WJ wrote the first draft of the manuscript. WJ, DH, JW, and JF finalized the submitted version of the manuscript.

### Conflict of Interest Statement

WJ has received research funding from Kin Master Indústrias Químicas, Passo Fundo, Brazil. The remaining authors declare that the research was conducted in the absence of any commercial or financial relationships that could be construed as a potential conflict of interest.
